# Insights into genome evolution, pan-genome, and phylogenetic implication through mitochondrial genome sequence of *Naegleria fowleri* species

**DOI:** 10.1038/s41598-022-17006-4

**Published:** 2022-07-31

**Authors:** Muhammad Aurongzeb, Yasmeen Rashid, Syed Habib Ahmed Naqvi, Hafiz Muhammad Talha Malik, Muhammad Kamran Azim, Syed Shah Hassan, Muhammad Yasir, Asad Karim

**Affiliations:** 1 Jamil-Ur-Rahman Center for Genome Research, Dr. Panjwani Center for Molecular Medicine and Drug Research, International Center for Chemical and Biological Sciences (ICCBS), University of Karachi, Karachi, 75270 Pakistan; 2 Department of Biochemistry, University of Karachi, Karachi, 75270 Pakistan; 3 Institute of Biotechnology and Genetic Engineering, University of Sindh, Jamshoro, Pakistan; 4 Alpha Genomics Private Limited, Islamabad, Pakistan; 5 Department of Biosciences, Mohammad Ali Jinnah University, Karachi, Pakistan; 6 Special Infectious Agents Unit, King Fahd Medical Research Center, King Abdulaziz University, Jeddah, 21589 Saudi Arabia; 7 Department of Medical Laboratory Technology, Faculty of Applied Medical Sciences, King Abdulaziz University, Jeddah, 21589 Saudi Arabia

**Keywords:** Genome informatics, Sequence annotation, Computational biology and bioinformatics, Genetics, Diseases, Pathogenesis

## Abstract

In the current study, we have systematically analysed the mitochondrial DNA (mtDNA) sequence of *Naegleria fowleri* (*N. fowleri)* isolate AY27, isolated from Karachi, Pakistan. The* N. fowleri* isolate AY27 has a circular mtDNA (49,541 bp), which harbours 69 genes (46 protein-coding genes, 21 tRNAs and 2 rRNAs). The pan-genome analysis of *N. fowleri* species showed a B_pan_ value of 0.137048, which implies that the pan-genome is open. KEGG classified core, accessory and unique gene clusters for human disease, metabolism, environmental information processing, genetic information processing and organismal system. Similarly, COG characterization of protein showed that core and accessory genes are involved in metabolism, information storages and processing, and cellular processes and signaling. The *Naegleria* species (n = 6) formed a total of 47 gene clusters; 42 single-copy gene clusters and 5 orthologous gene clusters. It was noted that 100% genes of *Naegleria* species were present in the orthogroups. We identified 44 single nucleotide polymorphisms (SNP) in the *N. fowleri* isolate AY27 mtDNA using *N. fowleri* strain V511 as a reference. Whole mtDNA phylogenetic tree analysis showed that *N. fowleri* isolates AY27 is closely related to *N. fowleri* (Accession no. JX174181.1). The ANI (Average Nucleotide Identity) values presented a much clear grouping of the *Naegleria* species compared to the whole mtDNA based phylogenetic analysis. The current study gives a comprehensive understanding of mtDNA architecture as well as a comparison of *Naegleria* species (*N. fowleri* and *N. gruberi* species) at the mitochondrial genome sequence level.

## Introduction

*Naegleria fowleri* (*N. fowleri*) is a habitant of warm lakes, streams, spas, pools, domestic water reservoirs and domestic water supplies^1–3^. *N. fowleri* species causes primary amoebic meningoencephalitis (PAM), which is an infection of the brain that results in the complete damage of brain tissue. Last year, between May 2021 to July 2021, a total of five PAM cases of brain-eating amoeba *N. fowleri* meningitis fatality have been reported in the megacity Karachi, Pakistan (https://www.dawn.com/news/1634761/another-naegleria-death-reported-in-karachi).

In protozoa, the organization of mitochondrial genomes (mtDNA) and their gene structure are more diverse compared to multicellular eukaryotes. *N. fowleri* mtDNA code for the major and minor rRNA components, some tRNAs, 46 proteins of the respiratory chain and ribosomes^4^.

A single genome sequence does not reflect all of a *N. fowleri* genetic diversity. A large number of genomic data is required for complex analyses such as molecular evolutionary and genomics pathogenesis. Fortunately, the ongoing advancement of sequencing technologies has helped in decreasing the time and cost in sequencing. As a result, there has been an exponential increase in the genomic databases. Comparative genomics, for example, is a new scientific topic that compares the genetic content of many taxonomically related microbes^5^.

Several alternative approaches, for instance, average nucleotide identity (ANI), can be used to assess taxonomic relationships in the post-genomic period^6^.

A pan-genome is a group of orthologous and unique genes found in a group of organisms. Pan and core genome analyses are critical for studying a phylogenetic lineage's, genomic and metabolic repertoires of an organism^7,8^. Although the pan-genome can refer to the whole collection of genes in a particular taxon, it is primarily defined for the species, which includes dispensable genes, all core genes and strain-specific genes^9–11^. Pan-genome analysis has been used to assess microorganism genomic diversity, evolution, pathogenicity, and other traits^12^.

Orthologous genes are groups of genes from distinct species that arose from a single ancestor gene, and generally these genes are involved in essential functions. The study of orthologous gene clusters in various strains might be useful to explore gene organization, gene function, and genome molecular evolution^13^. Furthermore, accurate recognition of orthologous genes helps in functional gene annotation, comparison, and evolutionary genomics research.

*Naegleria gruberi* (*N*. *gruberi*) is a non-pathogenic and non-thermotolerant closely relative of *N. fowleri*. So, a comparative genomics-based study could reveal new information about why *N. fowleri* causes such severe and fatal disease. Specifically, the genetic elements that give *N. fowleri* pathogenetic characteristics can be explored using a comparative genomics approach.

There are few data available on mitochondrial genome features and the evolution of *Naegleria* species. Only six complete mitochondrial genome sequences were publicly available till 1st August 2021 in the NCBI database.

In the current study, our main objective is to extend information on the mitochondrial genome of these species. We performed the complete mtDNA sequencing of *N. fowleri* isolate AY27 (Accession no. MZ461463), and studied the phylogeny and diversity using a comparative genomics approach. To the best of our knowledge, this is the first data on the characterization of mtDNA of *Naegleria* species based on ANI value, orthologous genes and pan-genome analysis. The findings of this study provide a comprehensive genetic landscape of the *Naegleria* species, as well as valuable insight into the protozoan's mtDNA.

## Materials and methods

### Patient sample

A cerebrospinal fluid (CSF) specimen number (AY-27) of 28 years-old male PAM patient was collected from “Karachi Diagnostic Center and Molecular Biology Laboratory” (https://www.kldc.pk/), approved by the Karachi Diagnostic Center and Molecular Biology Laboratory Ethical Committee (EC Ref No. REC-NF02). All methods were carried out following ethical regulations. The patient’s informed consent was obtained for CSF sample collection.

### Sample collection and identification

A direct microscopic examination was performed under a compound microscope (version n trademark) at 40 × after the CSF sample was incubated at 37 °C for 30 min. The sediments in the CSF sample were gently re-suspended in residual supernatant after centrifugation for 10 min at 250×*g*. Under sterile conditions 2 to 3 drops of re-suspended media was inoculated onto the Non-Nutrient Agar (NNA) plates covered with PAGE amoeba saline suspension containing *E. coli* ATCC25922.

The NNA cultured plates were covered using parafilm and incubated for 10 days at 42 °C^14,15^. Differentiation of *N. fowleri* from other *Naegleria* species based on cellular morphology is not simple. Generally, PCR-based identification is used for identification of *N. fowleri* from other *Naegleria* species^16–19^.

PCR product amplification was carried out in a total volume of 25 μl, containing 9.5 μl ddH_2_O, 0.5 μl primer (10 μM), 10 μl Green Master Mix (Promega, USA), and 5.0 μl genomic DNA isolated from CSF samples^18–21^. For PCR (40 cycle), the initial denaturation at 95 °C for 5 min, denaturation at 95 °C for 3 s, annealing at 53 °C for 30 s, extension at 72 °C for 30 s and the final extension at 72 °C for 5 min. The PCR product was visualized in a 2% agarose gel^16^.

We used two pairs of *N. fowleri* species-specific primers for the identification of *N. fowleri;* NaeglF192 (5′-GTGCTGAAACCTAGCTATTGTAACTCAGT-3′) and NaeglR344 (5′-CACTAGAAAAAGCAAACCTGAAAGG-3′)^20^; Nae3-F (5′-CAAACACCGTTATGACAGGG and Nae3-R TGGTTTCCCTCACCTTACG-3′)^21^.

### Library preparation and sequencing

The genomic DNA of trophozoite was isolate using commercially available DNA extraction kit (QIAmp DNA Mini Kit, QIAGEN). The concentration of DNA was calculated using Qubit 2.0 fluorometer (Invitrogen, ThermoFisher Scientific, USA). The genomic DNA library was prepared using the NEBnext Ultra kit (Illumina, San Diego, CA) and the Illumina platform (HiSeq 4000) was used for sequencing.

### Quality assessment, assembly, and gene annotation

FastQC was used to check the quality of the reads and the raw reads were pre-processed and the adapters were trimmed using Trimmomatic version 0.39^2^. The mtDNA was assembled in two steps: first, the reads were mapped onto the reference mtDNA (Accession Number: KX580903.1) using BWA tool^22^ and then spades software^23^ was used to assemble the mapped reads. To fill the gaps, we used Geneious Prime software (https://www.geneious.com/prime/). The quality of the assembly was checked using Quast software^24^. The mtDNA was annotated using the Pokka annotation tools^25^.

The GenBank file was used for graphical representation of BLAST results for DNA vs DNA and CDS vs CDS at cgview service (http://cgview.ca/viewer) and GenomeVX service (http://wolfe.ucd.ie/GenomeVx/) was used to plot the genomic features of *N. fowleri * isolate AY27

### Third-party sequencing data

We also used sequencing data of *Naegleria* species submitted to NCBI database (till 1st August 2021) by other research groups for comparative genomics. The details of *Naegleria* species used in the current study are given in supplementary Table S1. This included two strains isolated from the USA (KX580902.1, KX580903.1) one strain from Canada (JX174181.1), and one strain from Pakistan (OD958694.1) and *N. gruberi* (AF288092.1) was used as an outgroup.

### Evolutionary relationships of taxa

The evolutionary distances were calculated via the maximum composite likelihood method^2^ and evolutionary studies were conducted using MEGA X^3^ software. ANI (average nucleotide identify) matrix values were calculated for *Naegleria* species using OTA software (https://www.ezbiocloud.net/tools/orthoani).

### Evaluation of BPGA features with *Naegleria* species

To evaluate the PAN genome, the complete mtDNA sequences of five strains of *N. fowleri* including *N. fowleri* strain V511 (KX580902.1), *N. fowleri* strain V419 (KX580903.1), *N. fowleri* (JX174181.1), and *N. fowleri* isolate AY27 (MZ461463) were used for the analysis.

These sequences were annotated using Pokka software^25^. The gbk files were used as an input file in the BPGA pipeline^26^. BPGA uses USEARCH as the default clustering tool and the clustering output is used to perform pan-genome analysis. We used this pipline with 99% sequence identity as the cut-off value for pan-genome analysis. We also used BPGA pipeline^26^ for clusters of orthologous groups (COGs) and Kyoto Encyclopedia of Genes and Genomes (KEGG)^27^ pathway determination.

### Orthologous clustering analysis

The OrthoVenn2 web platform^28^ was used for orthologous clustering analysis of the protein-coding genes among *Naegleria* species. Orthofiner^29^ was used for calculation of overall statistics about orthogroups sizes and proportion of genes assigned to orthogroups.

### Nucleotide sequence accession number

The whole mtDNA sequence of *N. fowleri* isolates AY27 was deposited at GenBank database (Accession no. MZ461463).

## Results and discussion

### Isolation and identification of *N. fowleri*

**Figure 1:**
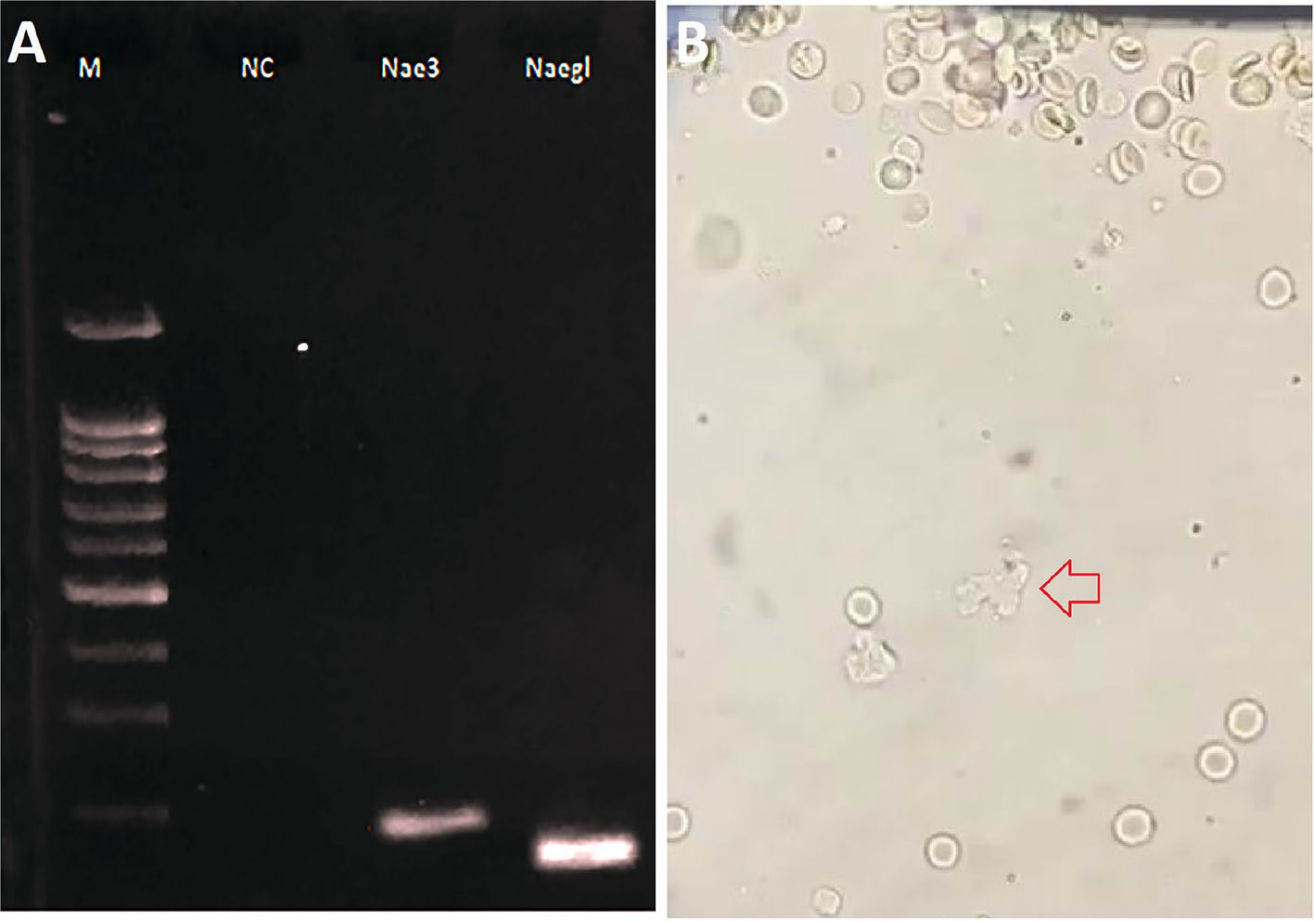
Agarose gel (2.0%) electrophoresis of the PCR products for analysis of patient CSF with primary amoebic meningoencephalitis (PAM) infection; line M: 100 bp DNA ladder, line NC: Negative control without DNA, line Nae3 (183 pb) and Naegl (153 bp): *N. fowleri* specific primers used for amplification of DNA from the patient CSF (**A**). Microscopy images: arrow indicating the *N. fowleri* observed in the patient CSF (**B**).

In the CSF sample (sample id = AY-27), direct microscopy (supplementary video 1: shows the motile forms of *N. fowleri* trophozoites in cerebrospinal fluid movement) revealed alive motile amoebic cells with pseudopodia (Fig. 1A). The trophozoite state of amoeba was suggested by the continuous change in cell morphology and formation of pseudopods (in sample AY-27). The trophozoites were about 12 to15 μm in size. Using eruptive pseudopods, the crawling amoeba was noted to move at a rate of ~ 1 µm/s. Selective identification of *Naegleria* species based on cellular morphology is difficult. Therefore, the PCR method was used for the detection of *Naegleria* species ^16–19^. We used two pairs of *N. fowleri* species-specific primers for the detection of *N. fowleri*. The amplified product size of Nae3-F_Nae3-F (Nae3) and NaeglF192-F_NaeglR344-R (Naegl) were found to be 183 bp and 153 bp, respectively (Fig. 1B, Supplementary Fig. S1).

### *N. fowleri* species mtDNA profile

**Figure 2:**
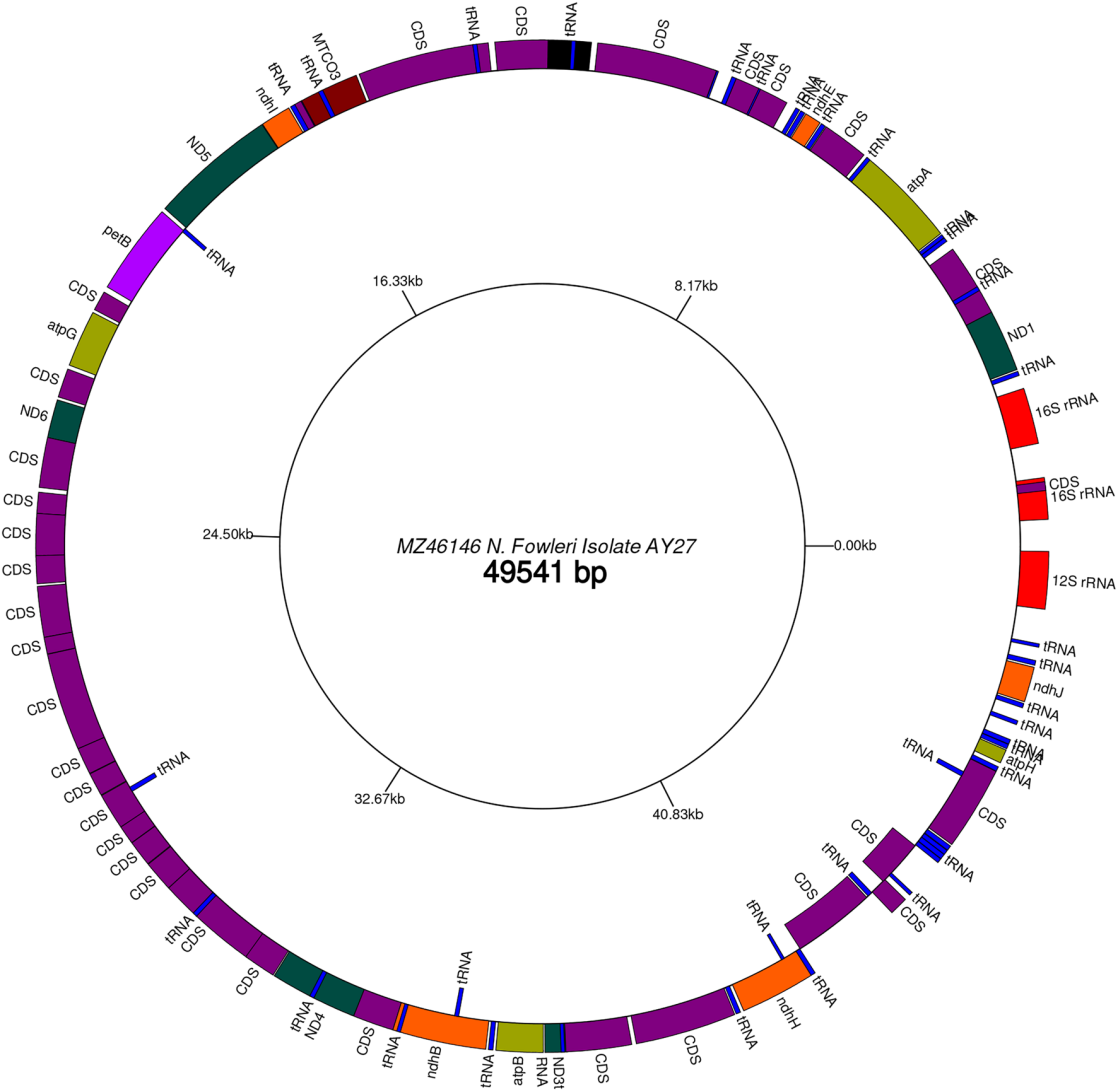
The complete mitochondrial of *N. fowleri* with GenomeVX service visualization shows the protein-coding genes, rRNAs, tRNAs and non-coding regions.

Figure 2 shows the mtDNA profile of *N. fowleri.* The size of mtDNA of our clinically isolated *N. fowleri* was found to be 49,541 bp.

The AT content of *N. fowleri* isolate AY27 is approximately 74.7%, while the GC content is 25.3%. The mtDNA of *N. fowleri* isolate AY27 consists of 69 genes (46 protein-coding genes, 2 rRNAs and 21 tRNAs). Among the protein-coding genes, 17 are ribosomal proteins (6 large and 11 small subunit). A total of 22 genes were found to encode various oxidative phosphorylation enzymes. Moreover, one copy of the tatC gene, which codes for the sec-independent translocase protein, one copy of the heme lyase gene, one copy of the ABC transporter subunit gene and four hypothetical proteins were also present (Supplementary Table S2).

### Evolutionary relationships of taxa

**Figure 3:**
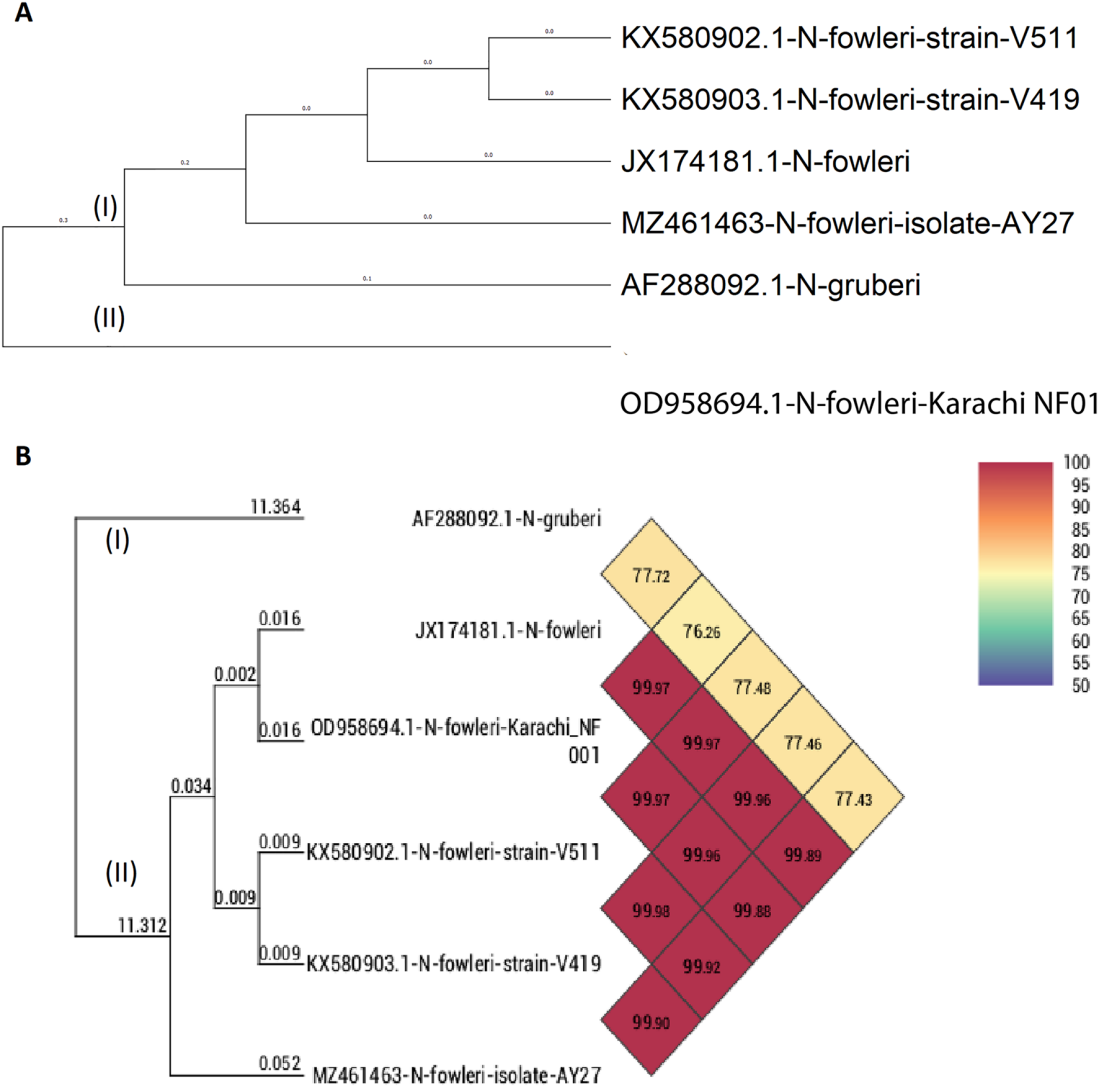
Whole mtDNA sequences were aligned and the phylogenetic tree was generated using the neighbour-joining method with 1000 bootstrap replicates (**A**) and heatmap showing the OrthoANI (Orthologous Average Nucleotide Identity) between *N. fowleri* isolate AY27 (MZ461463)) and other *N. fowleri* isolate calculated with OAT software, and *N. gruberi* species (AF288092.1) used as the outgroup (**B**).

This analysis involved six whole mtDNA sequences; *N. gruberi* (AF288092.1), *N. fowleri* Karachi NF001 (OD958694.1), *N. fowleri* strain V511 (KX580902.1), *N. fowleri* strain V419 (KX580903.1), *N. fowleri* (JX174181.1) and *N. fowleri* isolate AY27 (MZ461463). The phylogenetic tree analysis reveals that there were two clades (Fig. 3A). Clad I consist of *N. fowleri* strain V511 (KX580902.1), *N. fowleri* strain V419 (KX580903.1), *N. fowleri* (JX174181.1), and *N. fowleri* isolate AY27 (MZ461463). *N. gruberi* (AF288092.1) was also present in clade I but in a separate subclade. Clad II consist of *N. fowleri* Karachi NF001 (OD958694.1); It is interesting to note that *N. fowleri* Karachi NF001 (OD958694.1) was distinct from the rest of the three *N. fowleri* strains. Our *N. fowleri* isolates AY27 isolated from Karachi, presented higher similarities with *N. fowleri* (JX174181.1) compare to other strains.

The average nucleotide identity (ANI) is a method for assessing genetic relatedness, therefore we calculated the ANI values for *Naegleria* species. Briefly, ANI value gives the average nucleotide identity of all genes shared among two genomes^30^. As shown in Fig. 3B, the ANI values presented a much clear grouping of the *Naegleria* species compared to the whole mtDNA based phylogenetic analysis. *N. gruberi* species present in clade I presented ANI values in the range of 76.26% to 77.72%. On the other hand, in clade II, all the *N. fowleri* species ANI values were in the range of 99.88% to 99.98%.

### Pan and core-genome analysis of *N. fowleri* species

**Figure 4:**
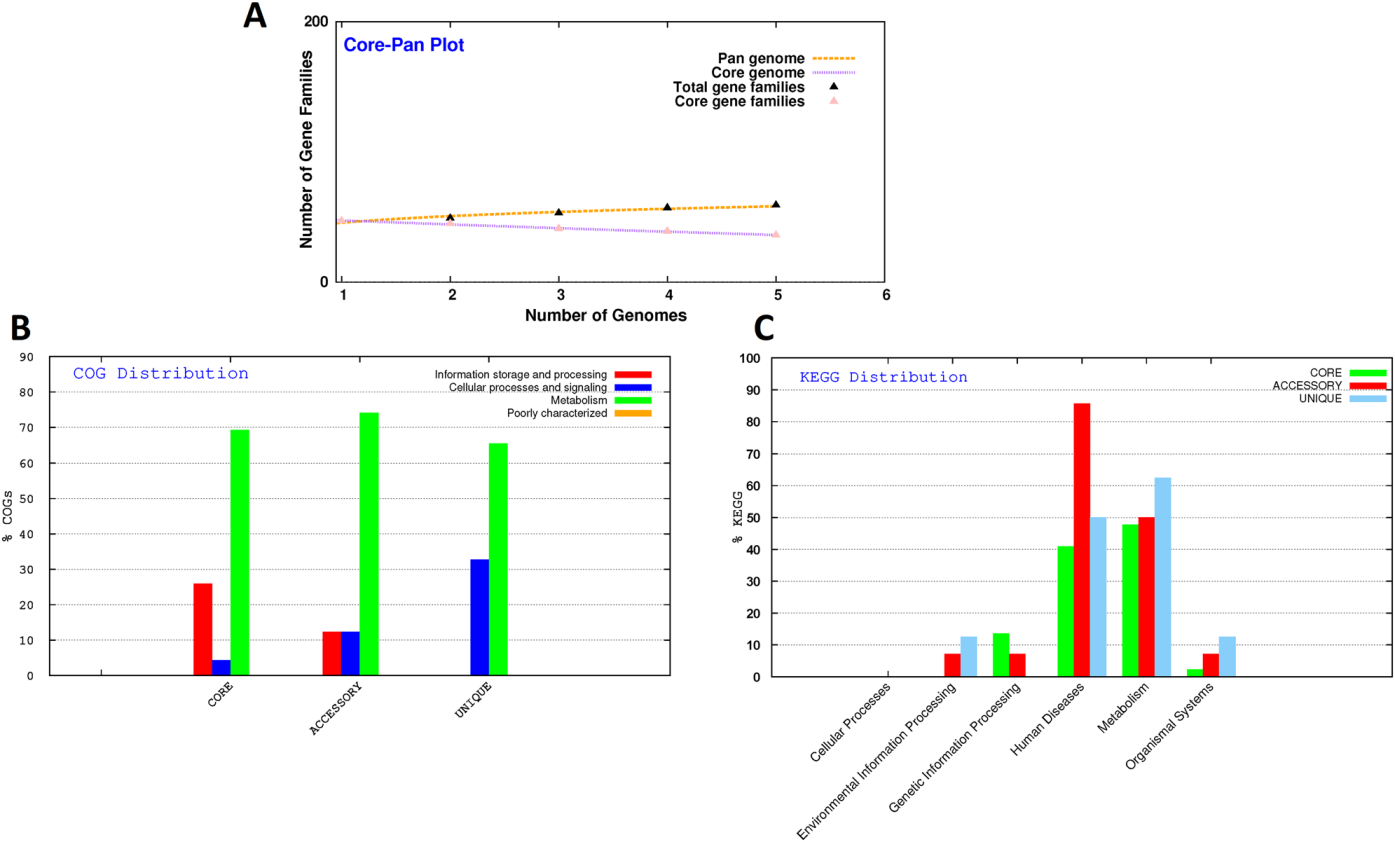
Pan-genome analysis (**A**), and COG (**B**) and KEGG (**C**) major category distribution of core, accessory and unique genes.

Figure 4A shows the pan-genome analyses of five *N. fowleri* species. The pan-genome of *N. fowleri* species showed a B_pan_ values of 0.137048 (i.e.,< 1) (Table 1), suggesting that the pan genome is still open but may be closed soon. This implies that for efficient environmental adaptations, the mtDNA is subjected to few regular evolutionary changes through gains and losses or lateral gene transfers. Thus, in the pan-genome, the number of gene families will continuously increase with the addition of new genomes to the analysis. Table 2 highlights the core, accessory, unique and exclusively absent genes. We identified 35 core genes in all five *N. fowleri* species. Moreover, all the five *N. fowleri* isolates have accessory genes and only *N. fowleri* strain V511 lacks both unique and exclusively absent genes.

**Table 1:** *Naegleria* species pan-genome overall statics.

Fit law	POWER	EXPONENTIAL
Equation	f(x) = a.x^b	f1(x) = c.e^(d.x)
Parameters	a = 0.137048	c = 48.5068
	*b = 0.137048	d = − 0.072729
Expected size	58	35
Estimated size	57.81	32.72

*The parameter B_pan_ (b) = 0.137048. The pan genome is still open but may be closed soon.

**Table 2:** *Naegleria* species pan-genome (core, accessory, unique and exclusively absent genes).

Genome no.	Organism name	No. of core genes	No. of accessory genes	No. of unique genes	No. of exclusively absent genes
1	JX174181.1	35	10	1	1
2	KX580902.1	35	11	0	0
3	KX580903.1	35	7	4	3
4	MZ461463	35	9	2	2
5	OD958694.1	35	9	3	3

### COG and KEGG distribution

COG classified all genomic sequences into three major groups; core, accessory and unique genes (Fig. 4B). We observed that core and accessory genes are involved in metabolism, information storages and processing, and cellular processes and signaling. Unique genes are associated to cellular processes and signaling, and metabolism. The details of COG distribution are given in Supplementary Figure S2; there are more (> 60%) core, accessory and unique genes for energy production conversion. Moreover, unique genes (30%) were present for post-translational modification. Similarly, accessory genes were also related to post-translational modification, protein turnover chaperones [O] and translation, ribosomal structure biogenesis [J].

The comparative KEGG distribution/details are plotted in Fig. 4C. The KEGG percentage shows core, accessory and unique gene clusters for human disease, metabolism, environmental information processing, genetic information processing and organismal system. Accessory genes were only involved in genetic information processing. Moreover, there are categories of unique genes for environmental information processing and genetic information processing. The details of KEGG distribution are given in Supplementary Figure S3. It is interesting to noted that core, accessory and unique genes were related to neurodegenerative diseases as well as endocrine and metabolic diseases.

### Core and pan phylogeny

**Figure 5:**
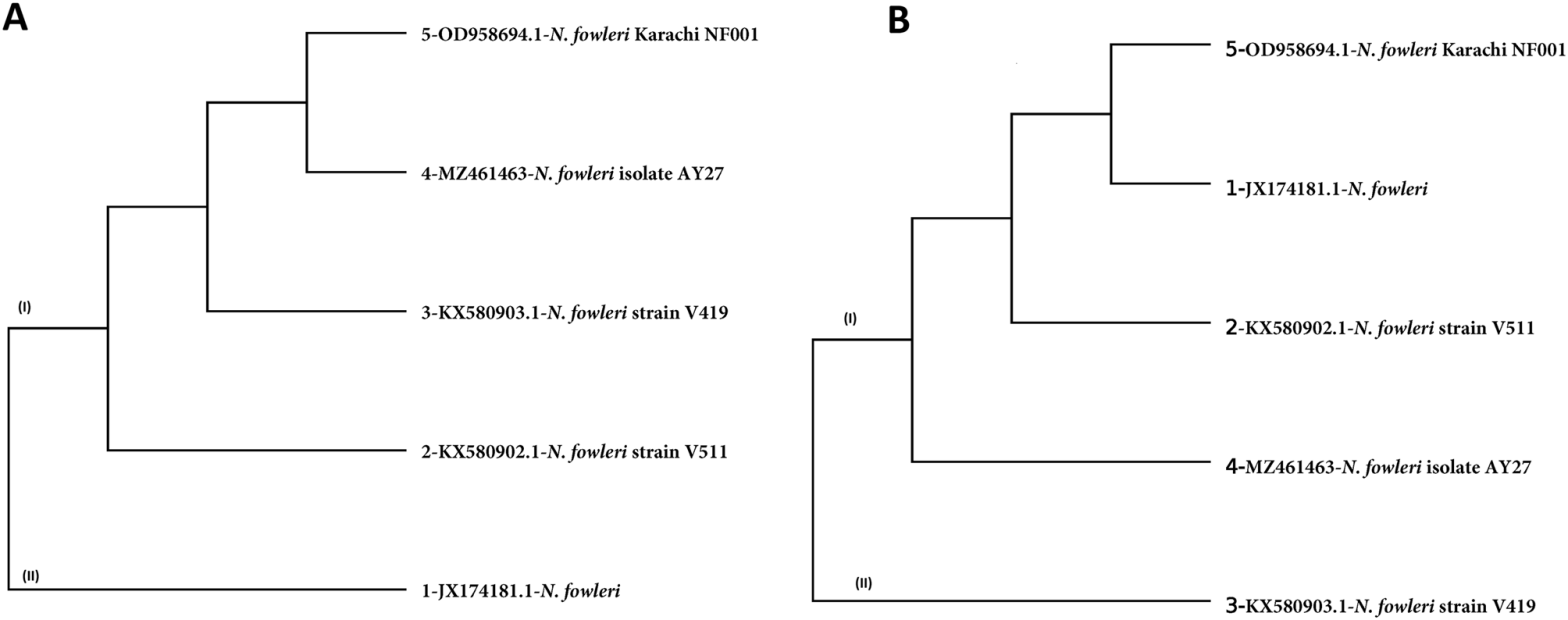
Phylogenetic analysis of core genome (**A**) and pan-genome (**B**) of *N. fowleri* species by BPGA pipeline.

The core (Fig. 5A) and pan phylogeny (Fig. 5B) indicates that just two groups initially arise from a single common ancestor. However, the first clad consist of four *N. fowleri* species and second clade consist of only one. In core and pan phylogeny, *N. fowleri* (JX17481.1) and *N. fowleri* strain v419(KX580903.1) were presented as a single separate group, respectively.

### Comparison of orthologous genes between the *Naegleria* species

Orthologous genes (277 genes) of *Naegleria* species (number of species 6) were analyzed and noted that 100% were present in the orthogroups (Table 3). The total number of orthogroups was found to be 46. The mean orthogroups size and median orthogroups size were found to be 6. Furthermore, number of single copy-orthogroups and number of orthogroups with all species present were 44 and 45, respectively. We also calculated orthogroups statistics for each *Naegleria* species and the details are given in Table 4.

**Table 3:** General statistics about orthogroups sizes and proportion of genes assigned to orthogroups.

Number of species	6
Number of genes	277
Number of genes in orthogroups	277
Number of unassigned genes	0
Percentage of genes in orthogroups	100
Percentage of unassigned genes	0
Number of orthogroups	46
Number of species-specific orthogroups	0
Number of genes in species-specific orthogroups	0
Percentage of genes in species-specific orthogroups	0
Mean orthogroup size	6
Median orthogroup size	6
G50 (assigned genes)	6
G50 (all genes)	6
O50 (assigned genes)	23
O50 (all genes)	23
Number of orthogroups with all species present	45
Number of single-copy orthogroups	44

**Table 4:** Statistics per species; orthogroups statistics for each *Naegleria* species.

*Naegleria* species	AF288092.1	JX174181.1	KX580902.1	KX580903.1	MZ461463.1	OD958694
Number of genes	46	46	46	46	47	46
Number of genes in orthogroups	46	46	46	46	47	46
Percentage of genes in orthogroups	100	100	100	100	100	100
Number of orthogroups containing species	46	46	46	46	46	45
Percentage of orthogroups containing species	100	100	100	100	100	97.8

**Figure 6:**
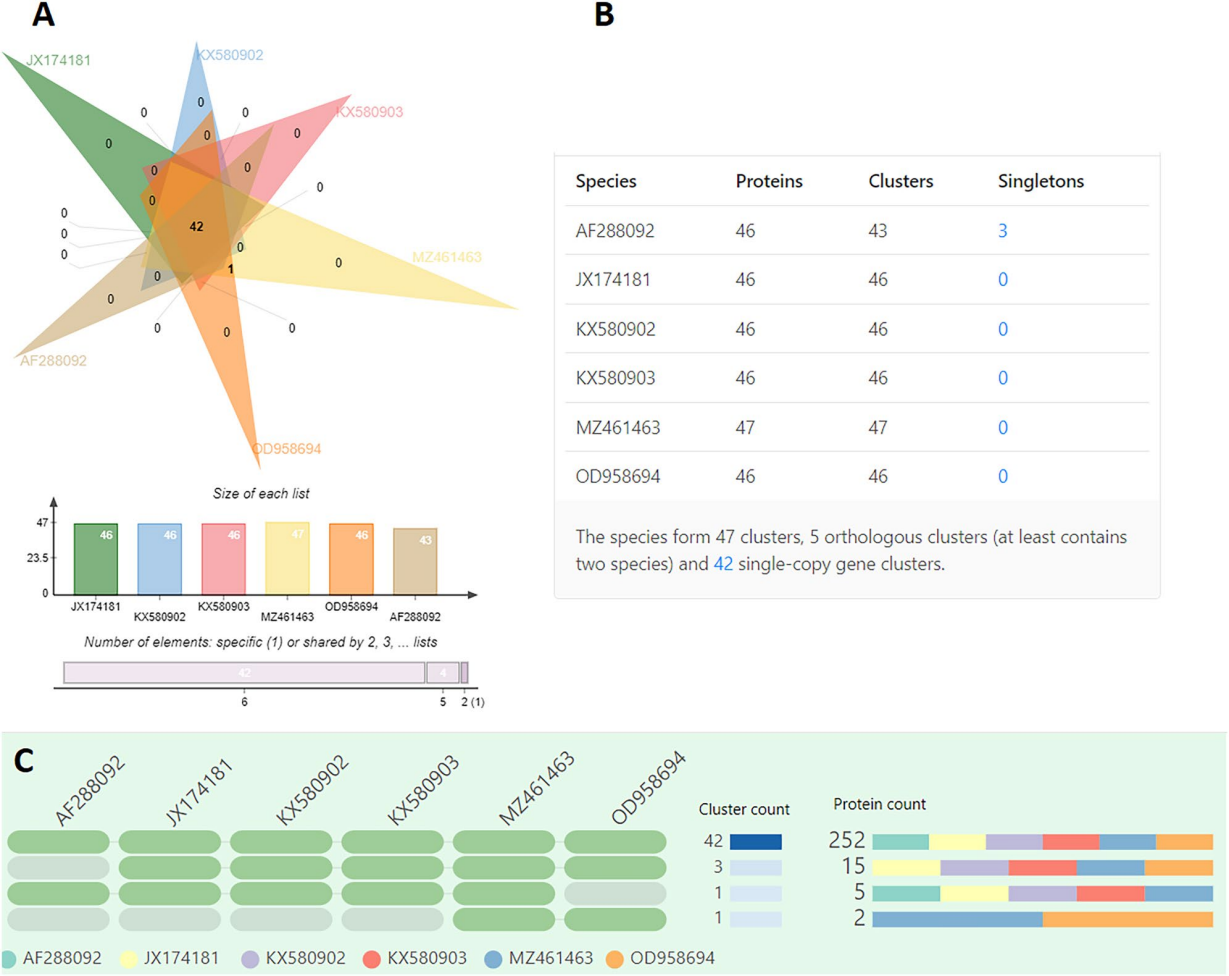
Venn diagram showing the distribution of orthologous gene clusters across *Naegleria* species and the total number of orthologous gene clusters of each organism (**A**). The orthologous cluster and singletons gene cluster (**B**). The occurrence table shows the occurrence pattern of shared orthologous groups among *Naegleria* species (**C**); the pattern towards the left shows the cluster count (the number of clusters shared between species) and protein count (number of protein members in the shared clusters).

The six *Naegleria* species shared 42 proteins (Fig. 6A). At the protein sequence level, the six *Naegleria* species form a total of 47 clusters; 42 single-copy gene clusters and 5 orthologous clusters (Fig. 6B). Three singleton genes (genes for which no orthologs could be found in any of the other species) were present in *N. gruberi* species (AF288092.1). Figure 6C shows the occurrence pattern of shared orthologous groups among five *N. fowleri* species*.* A green cell represents the presence of a cluster group in the corresponding species, and a grey bar represents the absence of a cluster group in that species. The pattern to the left shows cluster count and protein count in the shared clusters. The Gene Ontology (GO) distribution of the *N. fowleri* species of the 42 shared protein are given in Supplementary Table S3.

### Comparison of *Naegleria* species whole mtDNA

**Figure 7:**
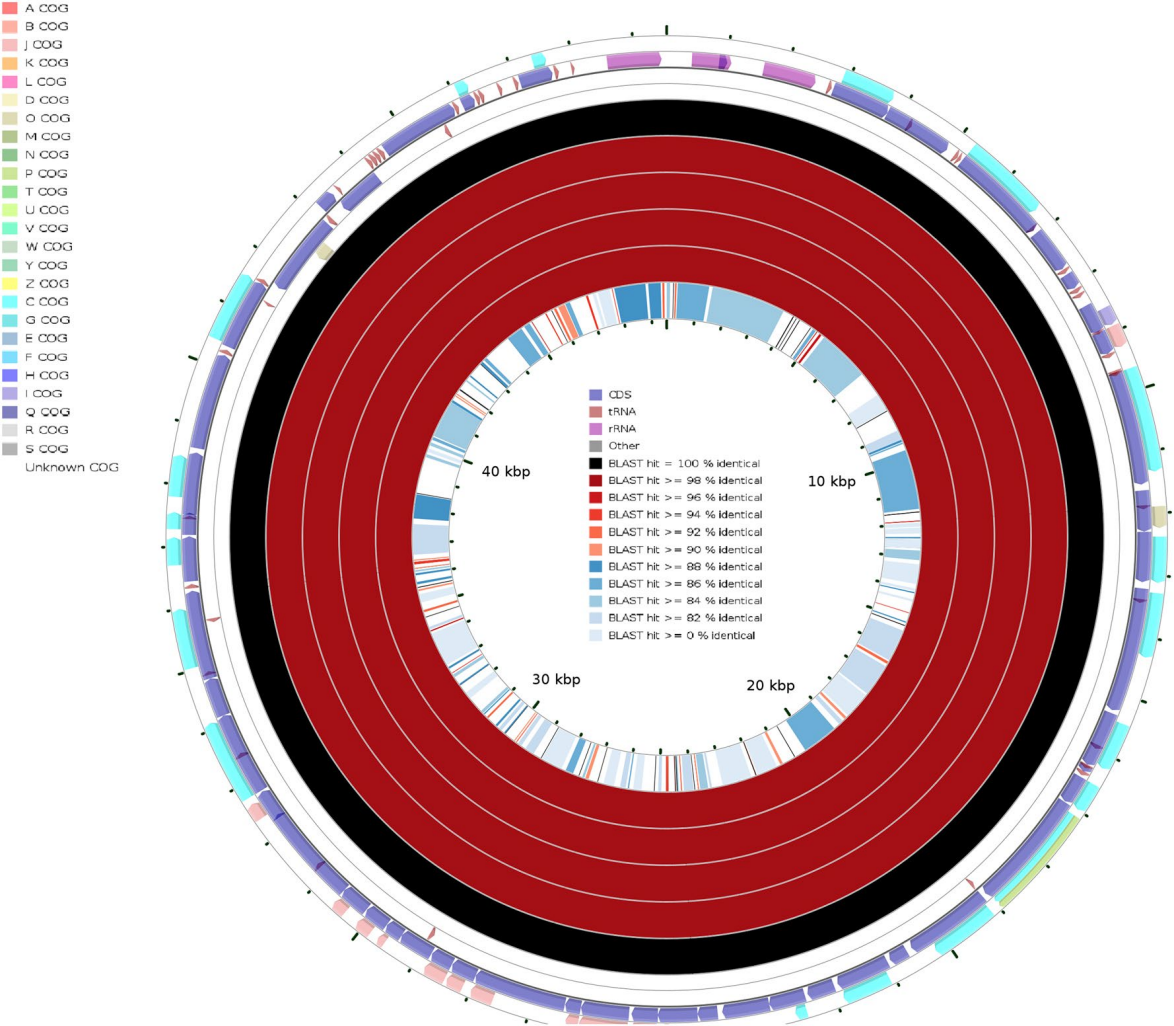
Graphical representation of BLAST results using *N. fowleri* isolate AY27 (MZ461463) mitochondrial DNA as a reference and other *Naegleria* species mitochondrial DNA as a queries. From the circle inward: the first ring represents the COG (Clusters of Orthologous Groups of proteins) grouping, second, third and fourth rings consist of CDS, tRNA, rRNA and other genes; fifth ring in black color represent the reference and six to nine rings are representing five *N. fowleri* species and the tenth ring is for *N. gruberi* species.

A comparative analysis of whole mitochondrial genomic DNA of *Naegleria* species was done using the cgview service. For this purpose, we used *N. fowleri* isolate AY27 (MZ461463) as a reference and the other five *Naegleria* species were taken as queries (Fig. 7)*.*

**Figure 8:**
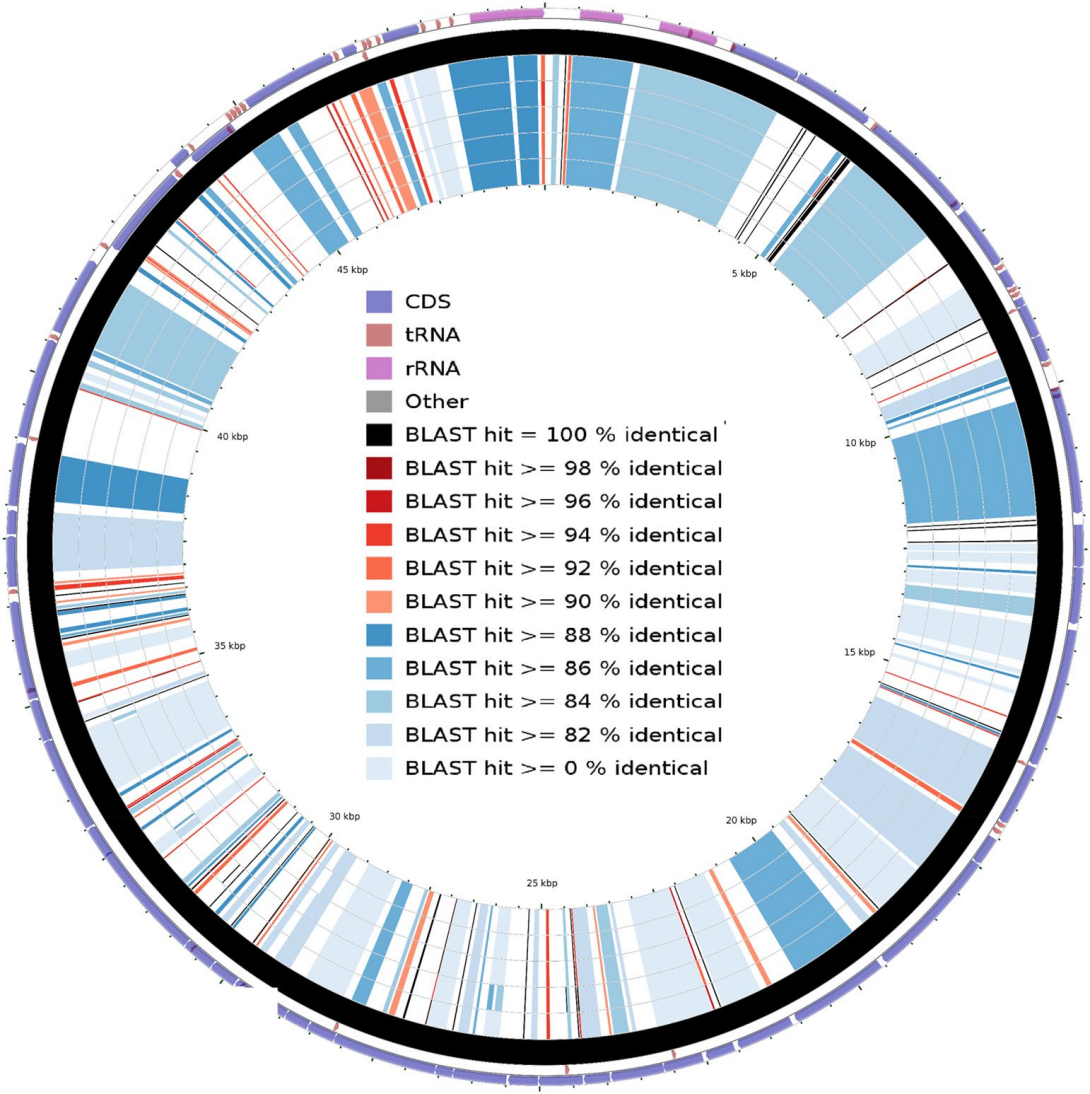
Graphical representation of BLAST results using *N. gruberi* species (AF288092.1) mitochondrial DNA as a reference and other *Naegleria* species mitochondrial DNA as a query. From the circle inward: the first and second rings consisting of CDS, tRNA, rRNA and other genes, third ring in black color represent the reference and six to eight rings are representing five *N. fowleri* species.

From the circle inward: the first ring represents the COG grouping, the second, third and fourth rings consist of CDS, tRNA, rRNA and other genes; fifth rings in black color represent the reference and six to nine rings are representing five *N. fowleri* species and the tenth ring is for *N. gruberi* species. These results portray that all the *N. fowleri* species were more than 98% identical.  As expected, when we used *N. gruberi* (AF288092.1) mtDNA as a reference and other five *N. fowleri* isolates as queries, the result showed that *N. gruberi* (AF288092.1) mtDNA was distinct from the *N. fowleri* species (Fig. 8). The result presented gaps/mismatches in the rings at several locations. However, at a few locations in the DNA, both *N. fowleri* species and *N. gruberi* species shared conserved regions.

Single nucleotide polymorphisms (SNPs) in mtDNA of *N. fowleri* isolate AY27 were analyzed using *N. fowleri* strain V511 as reference. We identified 43 SNPs in the *N. fowleri* isolate AY27 mtDNA (Supplementary Table S4). Most of the SNPs were present in the rRNA-large subunit ribosomal RNA gene. SNPs were also present in ATP1, ORF145, NAD4 and tRNA-Lys genes. Moreover, in the non-coding region, three SNPs were identified.

## Conclusion

The *N. fowleri* isolate AY27 mtDNA sequence has circular DNA with 69 genes and out of which 46 are CDS. The pan-genome analysis of *N. fowleri* species presented an open pan-genome characteristic. The core, accessory and unique gene were linked to human disease (> 40%). Orthologous gene analysis revealed that most of the mtDNA was conserved in the *Naegleria* species. We identified several SNPs in the *N. fowleri* isolate AY27 mtDNA using *N. fowleri* strain V511 as a reference. The data generated in the current study will help to understand these two species at the mitochondrial level.

## Supplementary Information

Supplementary Legends.

Supplementary Figure S1.

Supplementary Figure S2.

Supplementary Figure S3.

Supplementary Table S1.

Supplementary Table S2.

Supplementary Table S3.

Supplementary Table S4.

Supplementary Video 1.
