# Supplementary material for: Insights into genome evolution, pan-genome, and phylogenetic implication through mitochondrial genome sequence of Naegleria fowleri species
Source: Sci Rep. 2022 Jul 31;12:13152. doi: 10.1038/s41598-022-17006-4 (PMC9339544; doi:10.1038/s41598-022-17006-4)
Supplement: Supplementary file 1 — Supplementary Legends. [file 41598_2022_17006_MOESM1_ESM.docx]

**Figure legends for Supplementary**

**Figure S1.**  Agarose gel (2.0%) electrophoresis of the PCR products for analysis of patient CSF with primary amoebic meningoencephalitis (PAM) infection (M: Marker, NC: Negative Control, N. fowleri specific primer: Nae3 and Naegl and NS: ITS region)

**Figure S2.**  The details of COG distribution of core, accessory and unique genes.

**Figure S3.** The details of KEGG distribution of core, accessory and unique genes.
