# Supplementary figures and images for: Insights into genome evolution, pan-genome, and phylogenetic implication through mitochondrial genome sequence of Naegleria fowleri species
Source: Sci Rep. 2022 Jul 31;12:13152. doi: 10.1038/s41598-022-17006-4 (PMC9339544; doi:10.1038/s41598-022-17006-4)

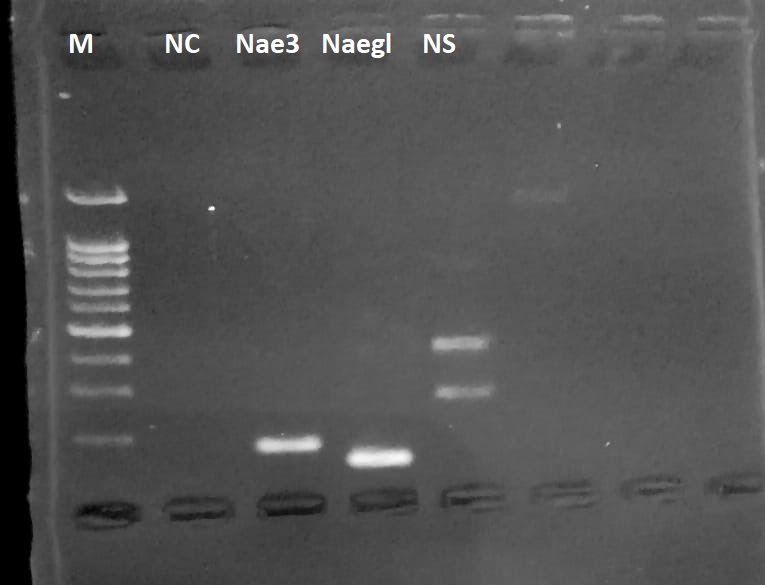

Supplement: Supplementary file 2 — Supplementary Figure S1. [file 41598_2022_17006_MOESM2_ESM.jpg]

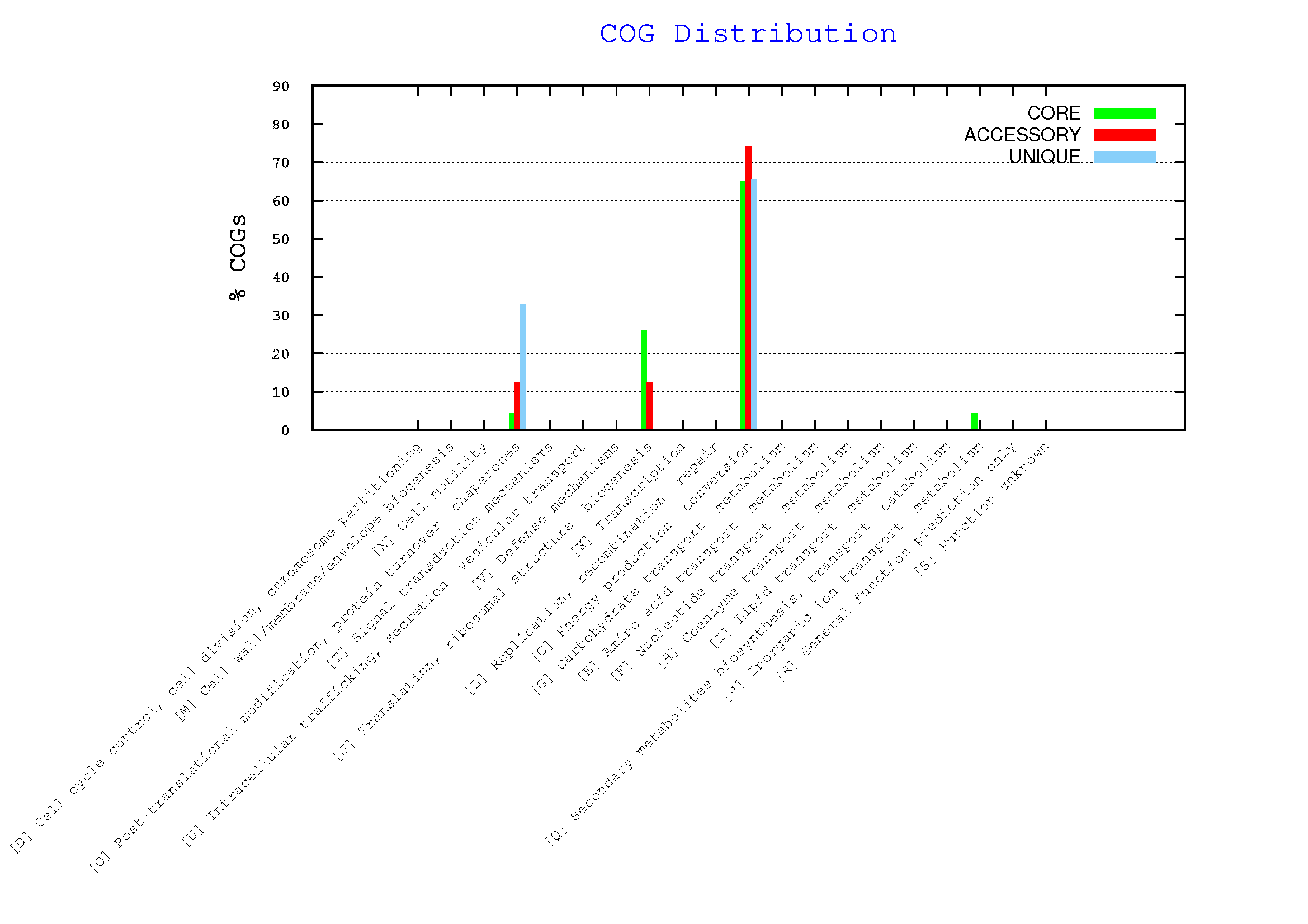

Supplement: Supplementary file 3 — Supplementary Figure S2. [file 41598_2022_17006_MOESM3_ESM.tiff]

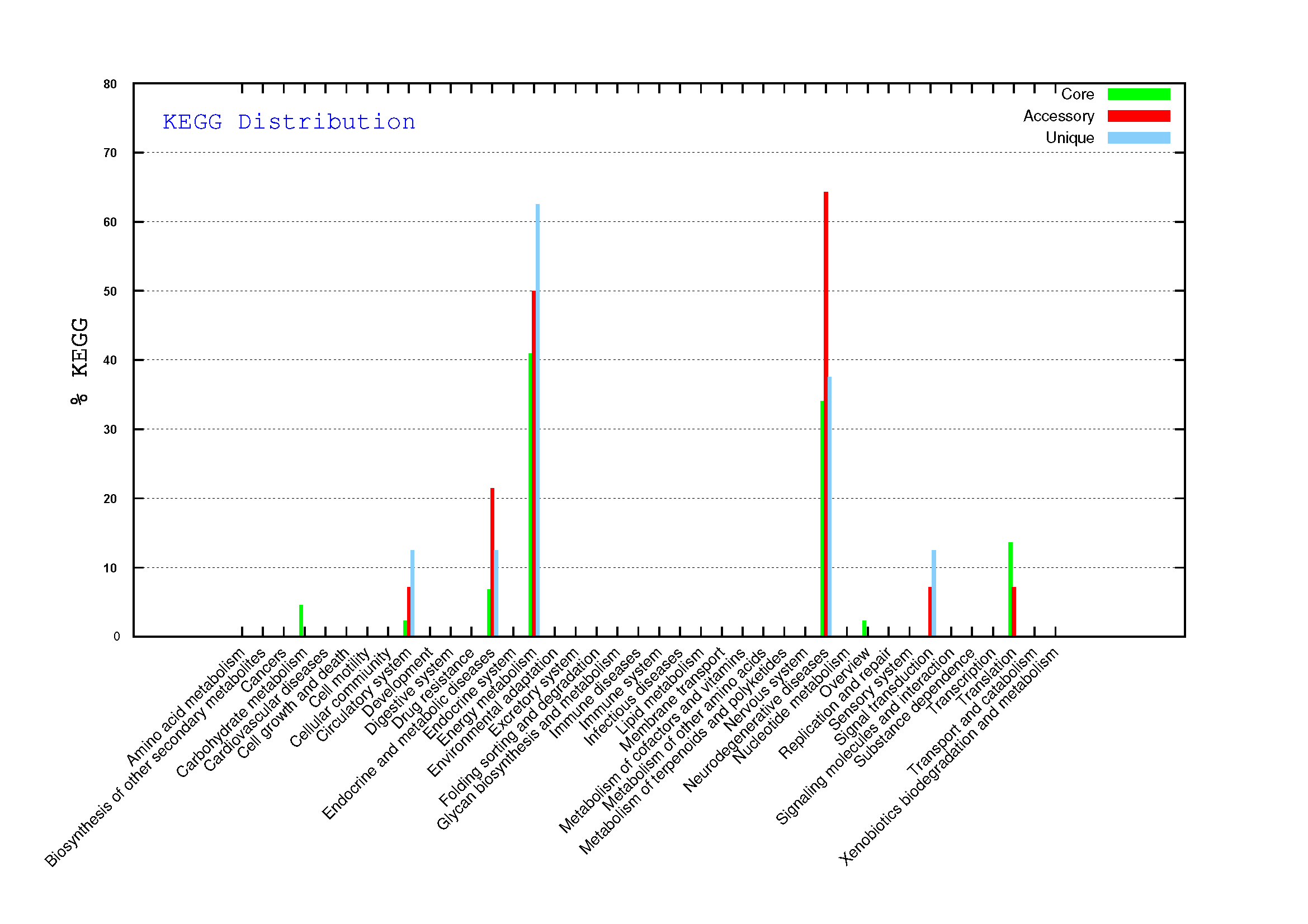

Supplement: Supplementary file 4 — Supplementary Figure S3. [file 41598_2022_17006_MOESM4_ESM.tiff]
