# Supplementary material for: Insights into genome evolution, pan-genome, and phylogenetic implication through mitochondrial genome sequence of Naegleria fowleri species
Source: Sci Rep. 2022 Jul 31;12:13152. doi: 10.1038/s41598-022-17006-4 (PMC9339544; doi:10.1038/s41598-022-17006-4)
Supplement: Supplementary file 5 — Supplementary Table S1. [file 41598_2022_17006_MOESM5_ESM.docx]

| **Accession No.** | ***Naegleria species*** | **Size**  **(bp)** | **G+C Content**  **(%)** | **Location** |
| --- | --- | --- | --- | --- |
| AF288092.1 | *N. gruberi* | 49843 | 22.2 | Canada |
| OD958694.1 | *N. fowleri* Karachi NF001 | 49836 | 25.2 | Karachi |
| KX580902.1 | *N. fowleri* strain V511 | 49541 | 25.2 | USA |
| KX580903.1 | *N. fowleri* strain V419 | 49538 | 25.2 | USA |
| JX174181.1 | *N. fowleri* | 49531 | 25.2 | Canada |
| MZ461463 | *N. fowleri* isolate AY27 | 49541 | 25.3 | Karachi |

**Table S1:** List *of Naegleria species* used in the current study**.**
