# Supplementary material for: Insights into genome evolution, pan-genome, and phylogenetic implication through mitochondrial genome sequence of Naegleria fowleri species
Source: Sci Rep. 2022 Jul 31;12:13152. doi: 10.1038/s41598-022-17006-4 (PMC9339544; doi:10.1038/s41598-022-17006-4)
Supplement: Supplementary file 6 — Supplementary Table S2. [file 41598_2022_17006_MOESM6_ESM.docx]

**Table S2:** Protein-coding genes in *N. fowleri* mitogenome

| **Categories** | **Sub-categories** | **Genes** |
| --- | --- | --- |
| Ribosomal proteins (17) | Small subunit (11) | Rps7,rps12, rps2, rps10, rps19, rps3, rps14, rps8,rps11, rps 13,rps4 |
|  | Large subunit (6) | Rpl11, rpl2, rpl16, rpl14, rpl5,rpl6 |
| Oxidative phosphorylation (22) | NADH dehydrogenase (11) | Nad1,nad4L nad11, nad8, nad5, nad6, nad4, nad2, nad3, nad7, nad9 |
|  | Ubiquinol:cytochrome C oxidoreductase | cob |
|  | Succinate:ubiquinone oxidoreductase | Sdh2 |
|  | Cytochrome c oxidase (4) | cox1,cox11,cox3,cox2 |
|  | ATP synthase (5) | atp1, atp3, atp8, atp6, atp9 |
| Sec-independent translocase protein (1) |  | tatC |
| hypothetical protein (4) |  | orf313,orf164,0rf145,orf504 |
| ABC transporter (1) |  | yeju |
| heme lyase (1) |  | yejR |
