# Supplementary material for: Insights into genome evolution, pan-genome, and phylogenetic implication through mitochondrial genome sequence of Naegleria fowleri species
Source: Sci Rep. 2022 Jul 31;12:13152. doi: 10.1038/s41598-022-17006-4 (PMC9339544; doi:10.1038/s41598-022-17006-4)
Supplement: Supplementary file 7 — Supplementary Table S3. [file 41598_2022_17006_MOESM7_ESM.docx]

| **Biological process summary** |
| --- |
| **Slimmed GO Name Count of unique input accessions** |
| GO:0006082 organic acid metabolic process 1  GO:0006091 generation of precursor metabolites and energy 6  GO:0006119 oxidative phosphorylation 6  GO:0006139 nucleobase-containing compound metabolic process 3  GO:0006412 translation 3  GO:0006725 cellular aromatic compound metabolic process 3  GO:0006793 phosphorus metabolic process 3  GO:0006807 nitrogen compound metabolic process 3  GO:0006810 transport 3  GO:0006811 ion transport 3  GO:0006818 hydrogen transport 3  GO:0008150 biological_process 10  GO:0008152 metabolic process 10  GO:0009116 nucleoside metabolic process 3  GO:0009117 nucleotide metabolic process 3  GO:0009987 cellular process 9  GO:0044237 cellular metabolic process 4  GO:0044238 primary metabolic process 4  GO:0045333 cellular respiration 7  GO:0046483 heterocycle metabolic process 3  GO:0051234 establishment of localization 3  **Molecular function summary**  GO:0009055 electron carrier activity 1  GO:0016491 oxidoreductase activity 2  GO:0048037 cofactor binding 1 |

**Table S3:** Gene Ontology (GO) distribution of the *Naegleria* species species proteins.
