# Supplementary material for: Insights into genome evolution, pan-genome, and phylogenetic implication through mitochondrial genome sequence of Naegleria fowleri species
Source: Sci Rep. 2022 Jul 31;12:13152. doi: 10.1038/s41598-022-17006-4 (PMC9339544; doi:10.1038/s41598-022-17006-4)
Supplement: Supplementary file 8 — Supplementary Table S4. [file 41598_2022_17006_MOESM8_ESM.docx]

**Table S4:** SNPs in mtDNA of *N. fowleri* isolate AY27 (MZ461463) using *N. fowleri* strain V511 (KX580902.1) as reference.

| **Sequence Position in MZ461463 (Query)** | **Query**  **Sequence** | **Position in KX580902.1 (Ref.)** | **Ref**  **Sequence** | **Gene** |
| --- | --- | --- | --- | --- |
| 431 | C | 431 | A | rRNA-large subunit ribosomal RNA |
| 450 | C | 450 | T |  |
| 730 | C | 730 | A |  |
| 736 | G | 736 | A |  |
| 1024 | T | 1024 | A |  |
| 1030 | A | 1030 | G |  |
| 1041 | C | 1041 | T |  |
| 1043 | C | 1043 | A |  |
| 1759 | T | 1759 | A |  |
| 1900 | G | 1900 | A |  |
| 2238 | G | 2238 | A |  |
| 2250 | T | 2250 | G |  |
| 2253 | G | 2253 | A |  |
| 2305 | C | 2305 | T |  |
| 2334 | G | 2334 | A |  |
| 2336 | C | 2336 | T |  |
| 2339 | - | 2339 | C |  |
| 2343 | T | 2343 | - |  |
| 2346 | G | 2345 | A |  |
| 2348 | C | 2347 | T |  |
| 2357 | A | 2356 | T |  |
| 2359 | G | 2358 | A |  |
| 2369 | C | 2368 | T |  |
| 2371 | T | 2370 | A |  |
| 2378 | A | 2377 | T |  |
| 2385 | C | 2384 | T |  |
| 2396 | T | 2395 | C |  |
| 2759 | C | 2758 | A | tRNA-Asn |
| 6895 | C | 6894 | A | atp1 |
| 6919 | T | 6918 | A |  |
| 22209 | T | 22208 | C | orf145 |
| 33681 | T | 33680 | G |  |
| 33699 | A | 33698 | C | nad4 |
| 46324 | A | 46323 | T | tRNA-Lys |
| 46325 | T | 46324 | C | Non-Coding gene |
| 46332 | G | 46331 | T |  |
| 46340 | T | 46339 | C |  |
| 48799 | G | 48798 | A | rRNA-small subunit ribosomal RNA |
| 48812 | G | 48811 | A |  |
| 48979 | C | 48978 | T |  |
| 48994 | C | 48993 | A |  |
| 48997 | C | 48996 | T |  |
| 49108 | C | 49107 | T |  |
